# Mucoadhesive Alginate/Pectin Films Crosslinked by Calcium Carbonate as Carriers of a Model Antifungal Drug—Posaconazole

**DOI:** 10.3390/pharmaceutics15102415

**Published:** 2023-10-03

**Authors:** Marta Szekalska, Anna Czajkowska-Kośnik, Bartosz Maciejewski, Iwona Misztalewska-Turkowicz, Agnieszka Zofia Wilczewska, Jurga Bernatoniene, Katarzyna Winnicka

**Affiliations:** 1 Department of Pharmaceutical Technology, Medical University of Białystok, Mickiewicza 2C, 15-222 Białystok, Poland; anna.czajkowska-kosnik@umb.edu.pl (A.C.-K.); kwin@umb.edu.pl (K.W.); 2 Department of Pharmaceutical Technology, Medical University of Gdańsk, Hallera 107, 80-416 Gdańsk, Poland; b.maciejewski@gumed.edu.pl; 3 Department of Polymers and Organic Synthesis, Faculty of Chemistry, University of Białystok, Ciołkowskiego 1K, 15-245 Białystok, Polandagawilczuwb@gmail.com (A.Z.W.); 4 Department of Drug Technology and Social Pharmacy, Faculty of Pharmacy, Medical Academy, Lithuanian University of Health Sciences, Sukileliu pr. 13, LT-50161 Kaunas, Lithuania; jurga.bernatoniene@lsmu.lt

**Keywords:** sodium alginate, pectin, posaconazole, crosslinking process, buccal film

## Abstract

The mucosal membrane of the oral cavity, due to its unique structure and availability, constitutes an appropriate site for the delivery of drugs, both with local and systemic effects. Mucoadhesive buccal films are drug dosage forms that due to their convenience of application, flexibility and size, are characterized by patients’ compliance. Sodium alginate and pectin are natural polymers from the polysaccharides group, with mucoadhesive properties, that are widely applied to obtain buccal films. However, their hydrophilic nature and poor water resistance limit their application in sustained drug release formulations. Hence, the aim of this investigation was to design alginate/pectin buccal films by a one-step crosslinking technique—with the application of calcium carbonate. This technique was applied to prepare crosslinked alginate and alginate/pectin mucoadhesive films with a model antifungal drug—posaconazole. The obtained formulations were evaluated for the impact of crosslinking and pectin’s presence on their pharmaceutical, mucoadhesive, mechanical and physicochemical properties. Additionally, the antifungal activity of the prepared films against *Candida* spp. was evaluated. It was shown that pectin’s presence in the formulations improved flexibility, mucoadhesion and antifungal activity. The crosslinking process reduced mucoadhesiveness and antifungal activity but significantly enhanced the mechanical properties and stability and enabled prolonged drug release.

## 1. Introduction

The most natural and also the most popular route of drug administration associated with drug absorption in the gastrointestinal tract is the oral route. However, there are certain limitations associated with the oral administration of drugs, for example, inactivation of the therapeutic substance or the first pass effect [1,2]. Therefore, other mucous membranes also deserve attention so that they can provide an alternative route of drug administration. The oral mucosa, due to the fact that it is easily accessible and located at the beginning of the digestive tract, provides the possibility for the application of the preparation to the required place and removal in emergency cases. The oral cavity area is the place where both topical and systemic active substances might be applied. Buccal administration of the drug avoids first-pass metabolism and its enzymatic degradation. In addition, it allows the use of an effective therapy for groups of patients with swallowing difficulties. As the oral cavity connects the external environment with the environment with the inside of the body, it can be the site of various pathologies that need topical treatment, such as oral candidiasis, periodontal disease, gingivitis, herpes or ulcers. However, this treatment can be significantly hampered by the brief adherence time of the drug form at the location of activity and the need for multiple doses throughout the day [3].

A key factor in the success of therapy for the treatment of conditions occurring in the oral cavity is the choice of the appropriate drug formulation. Particularly noteworthy are systems based on mucoadhesive polymers. Due to their interaction with the oral mucosa, they can be detained within the buccal cavity, thus extending the connection time of the drug form with the mucosa and allowing sustained release of the substance. Among the various solid dosage forms, mucoadhesive buccal films are supposed to be a suitable drug formulation because of their simplicity and convenience of application, flexibility and optimal size [4].

Sodium alginate (ALG) belongs to the group of natural polysaccharide polymers. Due to ALG’s advantages such as safety of application, non-toxicity, biodegradability and biocompatibility, polymer is often used in drug formulation technology. Chemically, it consists of β-D-mannuronic (M) and α-L-guluronic (G), which are associated by β (1→4) glycosidic bonds. Due to the presence of carboxyl groups, which can interact with hydroxyl groups of mucin glycoproteins, ALG is classified as an anionic mucoadhesive polymer. In addition, ALG possesses gelling properties. Despite the many significant advantages of ALG, ALG-based drug delivery systems also have limitations, such as low flexibility, poor mechanical properties and brittleness. Therefore, new solutions to improve the characteristics of ALG formulations are needed [5,6]. In our previous study, ALG buccal films with the addition of ALG oligosaccharides containing posaconazole (POS) as a model antifungal active substance were designed. Films were obtained by a relatively new freeze–thaw technique. The received data showed that films composed of pure ALG were characterized by poor mechanical properties: they were rigid, stiff and inflexible. It was observed that the addition of ALG oligosaccharides significantly improved their pharmaceutical quality [7]. Therefore, the idea of testing the influence of other natural polymers on the pharmaceutical properties of ALG buccal films seemed to be interesting.

Pectin (PEC), similar to ALG, belongs to the group of natural polysaccharides with mucoadhesive, swelling and gelling properties. PEC occurs naturally in the cell walls of various plant species. The structure of the molecule constitutes a homogalacturonan backbone with rhamnogalacturonan branches. The PEC homogalacturonan backbone consists of α-D-galacturonate residues linked by α (1 → 4) glycosidic bonds. PEC’s main component, galacturonic acid, might be methoxylated or amidated. In accordance with methoxylation degree (DM), PEC is ranked into two groups: high methoxy PEC (DM value of >50%) and low methoxy PEC (DM value of <50%). A polymer is often applied alone or in combination with other polymers in the development of various drug formulations [8,9].

ALG and PEC are polymers that are widely used to obtain buccal films. However, due to the hydrophilic nature of the polymers and poor water resistance, their application in extended-release drug forms is limited. It is known that ALG and PEC possess the ability to create water insoluble gels with divalent cations, and this fact might be used to improve these imperfections [10]. Therefore, the aim of this study was to prepare mucoadhesive ALG/PEC buccal films crosslinked by using calcium carbonate and glucono-δ-lactone with posaconazole (POS) as a model antifungal drug. POS is a broad-spectrum antifungal substance belonging to second-generation triazoles, for which the mechanism of activity includes ergosterol inhibition synthesis in the fungal cell wall [11]. POS application mainly includes the prevention of invasive *Aspergillus* and *Candida* infections in patients with immunodeficiency diseases or cancers, and the treatment of oropharyngeal candidiasis, especially in cases of resistance to itraconazole and fluconazole. POS is also used in the alternative treatment of invasive aspergillosis and infections caused by *Mucormycetes* and *Fusarium* spp., which are characterized by resistance to the treatment or in patients with intolerance to other antifungal drugs [12]. The application of calcium carbonate and glucono-δ-lactone is a new one-step crosslinking method used to obtain buccal ALG and ALG/PEC films, which leads to a slow gelation process of polymers. The prepared formulations were evaluated for the impact of the crosslinking agent on the pharmaceutical properties of ALG and ALG/PEC films. The mechanical, swelling and mucoadhesive qualities and the drug release profile of all formulations were also tested. A valid step of the investigation was to evaluate the antifungal action of the ALG/PEC films against more common *Candia strains*: *Candia albicans*, *Candia krusei* and *Candia parapsilosis*. Moreover, interactions between ALG, PEC and other applied excipients were evaluated via thermal analysis (thermogravimetric and differential scanning calorimetry analyses) and attenuated total reflectance–Fourier transform infrared spectroscopy.

## 2. Materials and Methods

### 2.1. Materials

ALG (procured from *Macrocystis pyrifera*) with medium viscosity (282 mPa∙s for 1% solution at 25 °C, 39% guluronic acid (G) and 61% mannuronic acid (M), characterized by molecular mass 3.5 × 10^5^ Da, M/G ratio of 1.56) was received from Sigma Aldrich (St. Louis, MO, USA). Calcium carbonate (CaCO_3_) and glucono-δ-lactone (GDL) were also purchased from Sigma Aldrich. Low methoxylated (LM) PECs (26% esterification, 24% degree of amination, 90% galacturonic acid content 90, 3.8 pH value of 2.5% solution prepared using distilled water at 20 °C) were kindly provided by Herbstreith & Fox and GmbH & Co. KG (Neuenbürg, Germany). POS was purchased from Kerui Biotechnology Co., Ltd. (Xi’an, China). Acetonitrile, methanol and polyethylene glycol (PEG 400) were procured from Merck (Darmstadt, Germany). Water was retrieved by the Milli-Q Reagent Water System (Billerica, MA, USA). Simulated saliva solution (SSS, pH 6.8) was obtained by using 8 g sodium chloride, 0.19 g potassium phosphate monobasic and 2.38 g of sodium phosphate dibasic per 1 L of water [13]. Sabouraud dextrose agar and cultures of *Candida albicans* ATCC^®^ 10231, *Candida krusei* ATCC^®^ 6528, *Candida parapsilosis* ATCC^®^ 22019 from the American Type Culture Collection were purchased from Biomaxima (Lublin, Poland). Cellulose acetate membrane filters (0.45 µm) were obtained from Millipore (Billerica, MA, USA), and nylon membrane filters (0.45 µm) were purchased from Alchem (Toruń, Poland). The procedure of obtaining a porcine buccal mucosa from a local slaughterhouse (Turośń Kościelna, Poland) did not need the agreement of the Local Ethical Committee for Experiments on Animals. Mucosa pieces were frozen at −20 °C prior to the investigation and were retained for up to one month. All other applied ingredients were of analytical grade.

### 2.2. Preparation of Non-Crosslinked Formulations

ALG solutions were received by the addition of a proper amount of polymer to water containing glycerol (GLY, performed the function of plasticizer), and they were mixed by a mechanical stirrer (DT 200, Witko, Łódź, Poland) to obtain a homogenous mixture. Then, into the ALG solution, PEC was added with continuous stirring. The content of non-crosslinked placebo formulations P1 and P4 is presented in Table 1. For non-crosslinked drug-loaded films, formulations F1 and F4 were prepared (Table 1). POS was solubilized in polyethylene glycol (PEG 400), and then the ALG/PEC solution was progressively placed in the mortar with the drug and the solubilizer. Then, the polymer solution was placed into plexiglass molds with a surface of 9 × 9 cm, retained in a fridge to eliminate air bubbles established in the mixing processes and then placed in the laboratory dryer at 40 °C for 24 h. Films were divided into parts of 2 × 2 cm by using a guillotine for paper (Fellowes, Doncaster, UK) with a total POS content of 2 mg/cm^2^.

### 2.3. Preparation of Crosslinked Formulations

The ALG/PEC solution was prepared according to Section 2.2. Then, calcium carbonate (CaCO_3_) and glucono-δ-lactone (GDL) was added into the polymer solution and mixed. The constant molar ratio of CaCO_3_:GDL was 0.5 and applied to maintain a neutral pH value [14]. The composition of the placebo formulations (P2, P3, P5, P6) is given in Table 1. For drug-loaded film formulations (F2, F3, F5, F6, Table 1), POS was solubilized in PEG 400 and the polymer solution was stepwise placed in the mortar containing the drug and the solubilizer. Then, the polymer solution was placed into plexiglass molds with a surface of 9 × 9 cm and placed in a fridge to eliminate air bubbles created in the mixing processes. Dried films were placed at 40 °C for 24 h and were divided into pieces of 2 × 2 cm by using a guillotine for paper (Fellowes, Doncaster, UK) with a total POS content 2 mg/cm^2^.

### 2.4. Viscosity Measurement

The viscosities of the performed non-crosslinked solutions and crosslinked hydrogels were measured 24 h after drafting via a Brookfield DV-III ULTRA Viscometer (Stuttgart, Germany). Measurements (with the shear rate of 2 s^−1^) of the hydrogel samples’ (0.5 g) viscosities were conducted in a temperature-controlled environment at 25 ± 1 °C using a CP-52 spindle.

### 2.5. pH Measurement

pH measurements of the prepared non-crosslinked solutions and crosslinked hydrogels were carried out at 25 ± 1 °C via the pH meter Orion 3 Star (Thermo Scientific, Waltham, MA, USA) equipped with a glass electrode.

### 2.6. Films Evaluation

#### 2.6.1. Scanning Electron Microscopy (SEM)

To assess the morphology of the prepared films, a scanning electron microscope (SEM) Phenom Pro G5 (Phenom World, Eindhoven, The Netherlands) was applied. Non-coated and sputter-coated with a 2 nm thick layer of gold film samples were imaged using an in-line detection mode at 5–10 kV with an 8 mm work distance and backscattered (BSD) detector.

#### 2.6.2. Weight and Film Thickness

The average weight of a minimum three appointed films was calculated to assess the films’ weight uniformity. Film thickness at six different locations was evaluated by a thickness gauge (Mitutoyo, Kawasaki, Japan).

#### 2.6.3. Moisture Presence

Moisture presence was conducted by applying moisture analytical balance (Radwag WSP 50SX, Radom, Poland).

#### 2.6.4. Homogeneity of Drug Content

Graduated flasks with films and 10 mL simulated saliva solution (SSS, pH 6.8) were placed in a water bath (50 rpm at 37 ± 0.5 °C). After 24 h of stirring, 20 mL of methanol was added. The prepared solutions were mixed, filtrated and evaluated by the HPLC technique (Section 2.6.5).

#### 2.6.5. High Performance Liquid Chromatography (HPLC) Analytics

The concentration of POS was examined by the HPLC technique applied using an Agilent Technologies 1200 system (Agilent, Waldbronn, Germany) equipped with a column Poroshell^®^ 120 EC-C18, which was characterized by a particle size of filling 2.7 μM, column diameter of 4.6 mm and length—150 mm (Agilent, Waldbronn, Germany). Acetonitrile, methanol and water were applied as the mobile phase in the ratio 60:20:20 (*v*/*v/v*) and a flow of 0.5 mL/min [11]. The analysis was performed with a wavelength of 240 nm. The standard calibration curve in the range of 1–100 μg/mL was linear with the correlation coefficient (R^2^) 0.999. POS expressed the retention time at 5.5 min.

#### 2.6.6. Disintegration Time

To assess the disintegration time of the prepared film formulations, two techniques were applied—in the apparatus used to evaluate disintegration time (Erweka ED-2L, Heusenstamm, Germany) and in a Petri dish. Determination of the disintegration time in conventional apparatus was prepare according to European Pharmacopoeia [15], and as a disintegration medium, 700 mL of simulated saliva solution (SSS, pH 6.8) was applied. To indicate the disintegration time using the second method, films were placed in the center of the Petri dish (with diameter 7 cm) containing 7 mL of SSS [16].

#### 2.6.7. Mechanical Properties

The mechanical characteristics of all prepared films, presented as tensile strength (TS), percent of elongation (E%) and Young’s modulus), were measured using the Texture Analyzer TA.XT. Plus (Stable Microsystems, Godalming, UK). After the preliminary tests, the parameters for the films’ evaluation were selected: the pretest, test and post-test speed was 1 mm/s, strain 10%, distance 3 mm and trigger force 0.001 N. The distance between the grips was 20 mm.

The experimental parameters of the process TS was computed by the equation:

TS = F/A
(1)

where F—applied stress and A—area.

E% was evaluated by the equation:

E% = [(L − L_0_)/L_0_] × 100 (2)

where L—film length after the elongation, L_0_—initial film length.

Young’s modu€ (E) was calculated by:

E = (F/A)/(L − L_0_/L) (3)

where F—applied stress, A—area of film, L_0_—initial film length and L—film length after the elongation.

Furthermore, each film was evaluated in terms of its folding endurance and presented as the number of folds before breaking [7].

#### 2.6.8. Swelling Properties

The swelling properties of the prepared films were presented as a swelling ratio (SR) determined using SSS (pH = 6.8). Accurately weighted films in the baskets (accessories from USP dissolution equipment) were situated in the beakers containing 15 mL SSS. Studies were conducted at the temperature of 37 ± 1 °C and at different time periods (5, 10, 15, 30, 45, 60, 90 and 120 min). After a certain time, the baskets were taken out of the beakers and cautiously strained. Furthermore, baskets containing films were weighted. The obtained data were applied to calculate the swelling ratio (SR) using the equation:

SR = (W_s_ − W_0_)/W_0_ (4)

where W_0_—initial film weight, W_s_—swelling film weight [17].

#### 2.6.9. Erosion Study

Accurately weighted films were placed in the Petri dish containing SSS pH 6.8 at 37.0 ± 0.5 °C. Films were retrieved from the media after 60 min and dried at 50 °C to a constant weight (W_d_). Erosion was computed based on the equation:

Erosion (%) = (W_0_ − W_d_)/W_0_ × 100, (5)

where W_0_—film initial weight, and W_d_—film weight after soaking in the SSS [17].

#### 2.6.10. Mucoadhesiveness

##### Ex Vivo Mucoadhesive Properties

The assessment of the mucoadhesive ability of the prepared films was performed by the TA.XT.Plus Texture Analyzer (Stable Micro Systems, Godalming, UK) and the porcine buccal mucosa was applied. Trial indicators of the test were: pretest speed of 0.5 mm/s, test and post-test speeds of 0.1 m/s, the time of contact was 180 s and force of 1 N. The mucoadhesive properties were expressed as the work of mucoadhesion (W_ad_) and the maximum detachment force (F_max_).

##### Ex Vivo Residence Time

The residence time test was performed by a technique known as the “wash off” study. It was applied using a modified USP disintegration tester equipped with a plexiglass cylinder (6 cm diameter, weight 280 g) moving vertically up and down. To the internal side of a beaker were glued the segments of porcine buccal mucosa (5 × 3 cm). The mucosal membrane with the film formulations were placed in the beakers containing 700 mL of SSS (pH 6.8) at a temperature of 37 ± 0.5 °C. The complete detachment of films from the mucosa was observed [18].

#### 2.6.11. In Vitro Drug Release

In vitro POS dissolution tests were performed by apparatus type II (Erweka Dissolution Tester Type DT 600HH, Heusenstamm, Germany) [15]. The paddle films were pasted using cyanoacrylate glue. The release investigation was carried out using 500 mL of SSS pH 6.8 with 1% sodium dodecyl sulfate (SDS, to obtain sink conditions) and a rotation speed of 75 rpm and temperature of 37 ± 0.5 °C. Sample of pure POS in the dose corresponding to its content in the evaluated films was used as a control. Drug concentration in the release medium was examined by applying the spectrophotometer Genesys 10S UV-Vis (Thermo Scientific, Madison, WI, USA). Analysis was carried out at the wavelength of 260 nm.

#### 2.6.12. Drug Release Mechanisms

To examine the POS release mechanism, results from the drug release experiment were evaluated using different mathematical models: zero order, first order, Higuchi, Korsmeyer-Peppas and Hixson-Crowell [19].

#### 2.6.13. Antifungal Activity Assay

Antifungal action of the prepared films was performed according to the Clinical and Laboratory Standards Institute (CLSI), and the plate diffusion technique was applied [20]. The initial density of *Candida* cells: *Candida albicans* ATCC^®^ 10231, *Candida krusei* ATCC^®^ 6528 and *Candida parapsilosis* ATCC^®^ 22019 was around 2–5 × 10^6^ colony forming units (CFU/mL). In the next step, the fungi inoculum in the sterile 0.9% NaCl solution was performed with the final density 0.5 in McFarland (5 × 10^4^ CFU/mL). The optical density of the inoculum was measured by using the suspension turbidity detector (Densitometer DEN-1B, Biosan, Riga, Latvia). A total of 50 µL of the *Candida* inoculum was placed on the Petri dishes with Sabouraud dextrose agar and rested for 15 min. In the next step, rings (with 5 mm diameter) of all film formulations were inserted on the surface of the plates. The POS, dissolved in DMSO, was used as the control. In this regard, 50 μL of solution of POS in DMSO (containing 1 mg of POS) was situated in a well in the agar basis with a 5 mm diameter. Samples were incubated for 24 and 48 h at a temperature of 37 ± 0.1 °C. Afterwards, the inhibition zones (mm) were labelled with a caliper (Mitutoyo, Kawasaki, Japan).

#### 2.6.14. Thermal Analysis

Thermal analysis was carried out applying thermogravimetric analyses (TGA) and differential scanning calorimetry analyses (DSC). Tests of the unprocessed substances: ALG, PEC, POS, CaCO_3_, GDL, film formulations without drug (P1, P2, P4, P5) and film formulations with POS (F1, F2, F4, F5) were evaluated by a Mettler Toledo Star TGA/DSC unit. To carry out the research performed by TGA and DSC, 3–5 mg weighted samples were deployed in aluminum oxide crucibles. Samples were heated in temperatures ranging from 25 °C to 480 °C with the speed at 10 °C/min under an argon flow and then cooled to 25 °C at a rate of −20 °C/min. One heating/cooling cycle under an argon flow rate of 200 mL/min was performed. As a reference, a blank pan was applied.

#### 2.6.15. Attenuated Total Reflectance–Fourier Transform Infrared Spectroscopy (ATR–FTIR)

ATR–FTIR spectra of the unprocessed ALG, PEC, POS, CaCO_3_, GDL, film formulations without drug (P1, P2, P4, P5) and film formulations containing POS (F1, F2, F4, F5) were received from a Thermo Scientific Nicolet 6700 FTIR spectrophotometer (Waltham, MA, USA) with diamond attenuated total reflectance. The spectra were analyzed with the background spectra, and 32 scans with a resolution of 4 cm^−1^ in the range between 500 cm^−1^ and 4000 cm^−1^ were taken.

#### 2.6.16. Statistical Analysis

The received results were evaluated applying Statistica 13.3 Software (TIBCO Software Inc., Palo Alto, CA, USA). The statistical study was examined by one-way analysis of variance (ANOVA) with a post hoc Tukey’s test or Kruskal–Wallis test. Quantity variables were presented as the mean and standard deviation.

The statistical significance level was set at less than 0.05 (*p* < 0.05). The three-dimensional (3D) response surface was retrieved from Statistica 13.3 Software to procure the optimum points of the ALG/PEC crosslinked hydrogels’ viscosity and film thickness.

## 3. Results and Discussion

**Figure 1:**
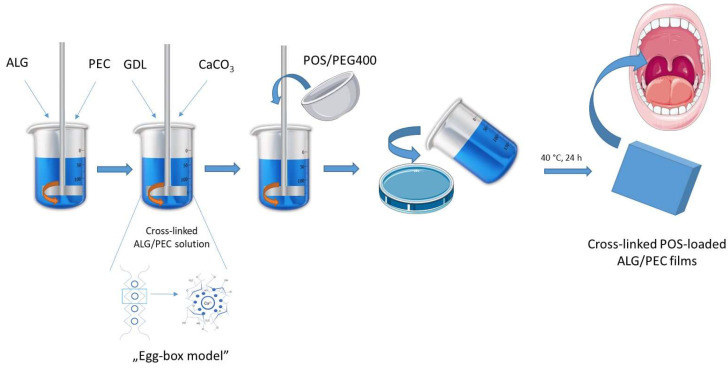
Scheme of preparation of crosslinked POS-loaded ALG/PEC films. Picture was prepared by using parts from Servier Medical Art, ensured by Servier, licensed under a Creative Commons Attribution 3.0 unported license [23].

ALG and PEC are among the natural polyuronates, with the most important feature being the ability to gel under the influence of divalent cations, e.g., Ca^2+^. The gelation mechanisms, widely known as the “egg-box model”, is the effect of strong and specific reaction between Ca^2+^ ions and guluronate blocks in ALG backbones and galacturonate blocks existing in PEC. Calcium chloride (CaCl_2_), the most frequently used donor of Ca^2+^ ions, leads to rapid and uncontrolled gelation of polymers. Extension of the gelation time allows the formation of uniform gel structures. Calcium carbonate (CaCO_3_) is a substitute for CaCl_2_, which dissociates due to excess hydrogen atoms. The application of GDL, the optimal dissociating agent, leads to a slow gelation process of polymers, which significantly depends on the values of temperature and pH. This fact enables the crosslinked ALG/PEC mixture to be poured into molds before gelation occurs [21,22]. Figure 1 introduces the scheme of preparation for crosslinked POS-loaded ALG/PEC films.

**Figure 2:**
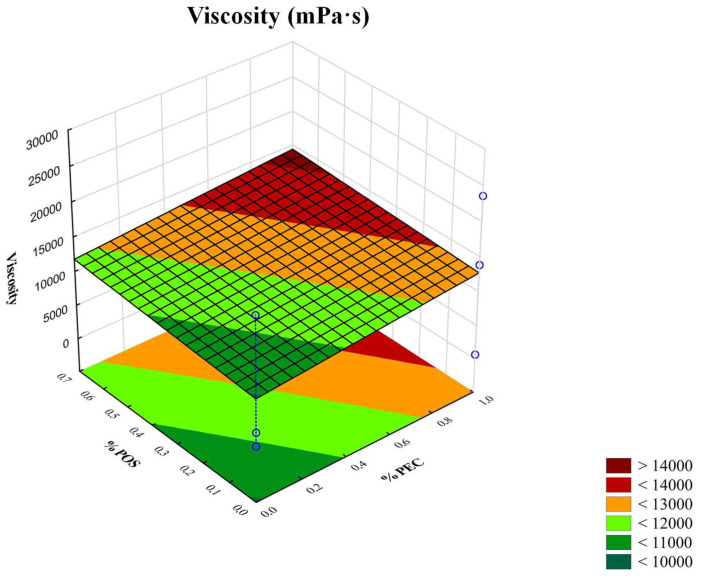
Three-dimensional (3D) response surface diagram of viscosity vs. PEC and POS concentrations.

As a result of preliminary investigations, 2% of the ALG solutions with advantageous viscosity were selected to prepare the films. Polymer solutions, which are characterized by insufficient viscosity, contributed to films being obtained that possessed low values of thickness and weak mechanical features. In turn, high viscosity precluded the casting solution on the plexiglass plate. In addition, due to the optimal viscosity values, two optimal concentrations of crosslinking agents, CaCO_3_ and GDL (Table 1) with a constant molar ratio of 1:2, were selected for the gelation process [14]. The viscosity values of the prepared non-crosslinked ALG/PEC solutions and crosslinked hydrogels are indicated in Table 2. The presented data demonstrate that the solutions composed only from ALG were characterized by a higher viscosity than solutions with ALG and PEC. It was observed that the crosslinking process significantly increased the viscosity of ALG and ALG/PEC gels. Interestingly, crosslinked ALG/PEC hydrogels possessed higher values of viscosity than crosslinked ALG hydrogels. The three-dimensional (3D) response surface diagram expresses that the addition of PEC and POS increased the viscosities of all hydrogels (Figure 2). Formulations composed of ALG and PEC have significantly lower pH values compared to formulations composed of pure ALG due to the acidic nature of PEC. The crosslinking agent’s presence insignificantly (*p* > 0.05) decreased pH values in formulations consisting of ALG only (from 7.82 ± 0.01 to 6.39 ± 0.02) and consisting of ALG and PEC (from 4.92 ± 0.01 to 4.77 ± 0.02). It was shown that the values of pH changes did not affect the viscosities of the ALG and ALG/PEC non-crosslinked solutions. Comparing the crosslinked formulations, it was observed that when the pH value decreased, the viscosities of the crosslinked formulations increased (Table 2).

### 3.1. Evaluation of Buccal Films

It was noted that the film formulation placebos, both non-crosslinked and crosslinked, were dry, soft and non-sticky. The prepared films were translucent and possessed a slight yellow tint from the ALG. The POS-loaded formulations, in turn, were characterized by a white color derived from POS. The pharmaceutical qualities of the designed buccal ALG and ALG/PEC films are expressed in Table 3.

**Figure 3:**
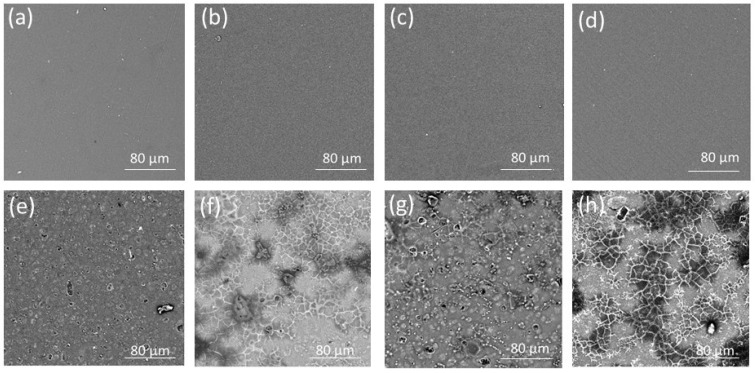
Surface representative images of non-crosslinked formulation placebos: (**a**) formulation P1, (**b**) formulation P4; crosslinked formulation placebos: (**c**) formulation P2 and (**d**) formulation P5; non-crosslinked POS-loaded formulations: (**e**) formulation F1 and (**f**) formulation F4; and crosslinked POS-loaded formulations: (**g**) formulation F2 and (**h**) formulation F5, under magnification ×1000.

**Figure 4:**
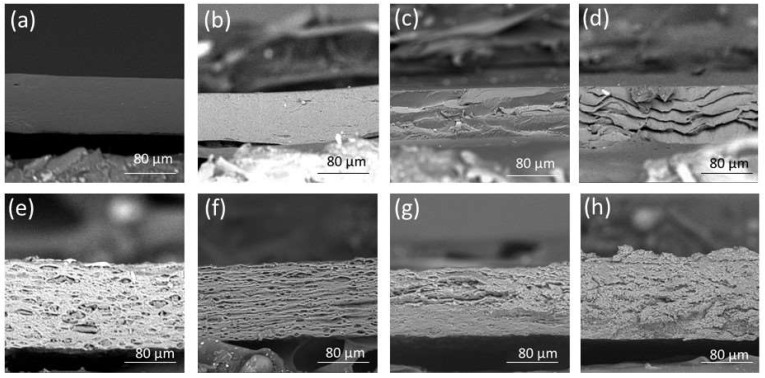
Cross-section representative images of non-crosslinked formulation placebos: (**a**) formulation P1 and (**b**) formulation P4; crosslinked formulation placebos: (**c**) formulation P2 and (**d**) formulation P5; non-crosslinked POS-loaded formulations: (**e**) formulation F1 and (**f**) formulation F4; and crosslinked POS-loaded formulations: (**g**) formulation F2 and (**h**) formulation F5, under magnification ×1000.

SEM analysis provides details about a film’s microstructure. The surface and cross-sectional SEM micrographs of the non-crosslinked and crosslinked film placebos and POS-loaded formulations are presented in Figure 3 and Figure 4. It was shown that the non-crosslinked and crosslinked film placebo formulations were characterized by a smooth and homogenous surface (Figure 3). However, POS-loaded films demonstrated a heterogeneous structure with gelled areas. The cross-section of the placebo non-crosslinked films expressed a homogenous nature. The crosslinked formulations were characterized by fibrous and porous structures with irregularities (Figure 4).

**Figure 5:**
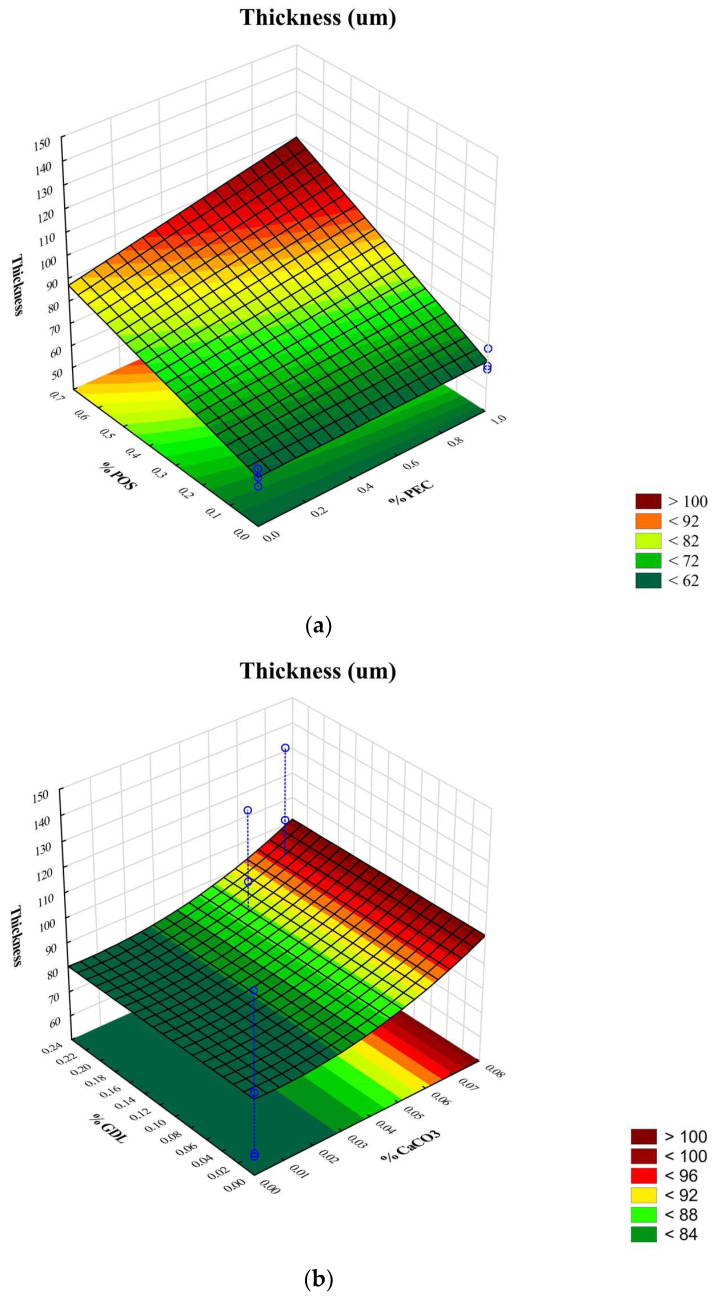
Three-dimensional (3D) response surface diagrams of (**a**) thickness versus PEC existence and POS concentrations and (**b**) thickness versus CaCO_3_ and GDL concentrations.

Moisture content is one of the crucial properties of films that significantly affects disintegration time. Preis et al. demonstrated that films characterized by high values of moisture content tend to disintegrate faster than films with a lower moisture content [16]. Water presence in the formulation is also relevant for flexibility closely associated with packaging possibility and application comfort. In turn, the high moisture content of the films can facilitate the growth of microorganisms and lead to recrystallization of the drug substance. In all designed film formulations, moisture presence, expressed as percentage values, obtained values from 4.11 ± 0.85% to 5.71 ± 1.20% (Table 3). The obtained values are within the range according to Borges et al., who reported that the moisture content in oral film should be in the range of 3–6% [24]. Weight uniformity is a key parameter, which determines the accuracy of the drug dosage. The obtained results show that the weights of the prepared films were in the range from 38.28 ± 3.43 for P1 to 57.88 ± 3.62 for F3. In general, the weight of the films increased with the presence of crosslinking factors and POS. The PEC addition insignificantly affected the change in the weight of films. Film thickness is an indicator that significantly affects the mechanical properties, disintegration time, swelling properties and drug release. The thicknesses found ranged from 57.67 ± 5.19 μm in P1 to 68.17 ± 5.42 in P6 and from 83.06 ± 9.31 in F1 to 139.22 ± 3.72 μm in F6 (Table 3). According to the three-dimensional response surface graph, it was shown that PEC and POS presence increased films thickness (Figure 5a). Additionally, it was expressed that crosslinked films were characterized by a significantly higher thickness (*p* < 0.05) (Figure 5b). Similar data for the increasing thicknesses of ALG/PEC films crosslinked with zinc ions prepared by the external gelation method were noted by Nešić et al. [25]. It was shown that the values of films thickness were significantly (*p* < 0.05) enhanced with POS presence and in the case of crosslinked formulation. However, the presence of PEC significantly (*p* < 0.05) changed the thickness of the films (Table 3).

As pH values deviating from physiological values might irritate the buccal mucosa, an important part of the study was to evaluate the pH values of the prepared hydrogels. The results obtained were within the optimal range, indicating compatibility with physiological buccal pH [17]. The pH measurement of obtained hydrogels indicated that both the presence of PEC in the formulation and the presence of crosslinking agents decreased the initial pH values. In addition, formulations containing POS showed slightly higher pH values than the corresponding placebo formulations. The values of surface pH ranged between 6.87 ± 0.02 and 6.87 ± 0.01 for formulations F5 and F6, respectively, and 6.94 ± 0.02 for formulation P1. The noted pH values were approximated to the pH of buccal mucosa (6.28) and prepared films were recognized as being appropriate for buccal administration [26] without a negative impact on the oral mucosa (Table 3). An important parameter of film quality is the evaluation of drug content uniformity, which according to the pharmacopoeial requirements should be in the range from 85% to 115% [13]. It was noticed that the presence of POS in all of the prepared formulations was in the range from 94.72 ± 4.01% to 106.39 ± 6.05%. The obtained results fulfilled the pharmacopoeial requirements and expressed that the experimental parameters did not affect the drug content uniformity (Table 3). Additionally, the received data expressed no significant disparities in POS content within a particular formulation (*p* > 0.05). This fact demonstrated that the presented technique was characterized by reproducibility and allows uniform distribution of the active substance in the films.

Disintegration time affects drug release, which is intensely related to the medium volume. Therefore, in this study, disintegration time was determined in the pharmacopoeial apparatus with 700 mL SSS and using a Petri dish containing 7 mL of the medium in order to simulate saliva capacity inside the human oral cavity (Table 4). It was observed that PEC and POS presence in the formulation had an effect on the extension of the disintegration time. It was reported that the time of the film’s disintegration increased with the increasing crosslinking agent’s concentration and with the reduced volume of the medium. The test conducted in the Petri dish expressed that the disintegration time was sustained, which is a desirable attribute for buccal administration [27].

### 3.2. Mechanical Properties

**Figure 6:**
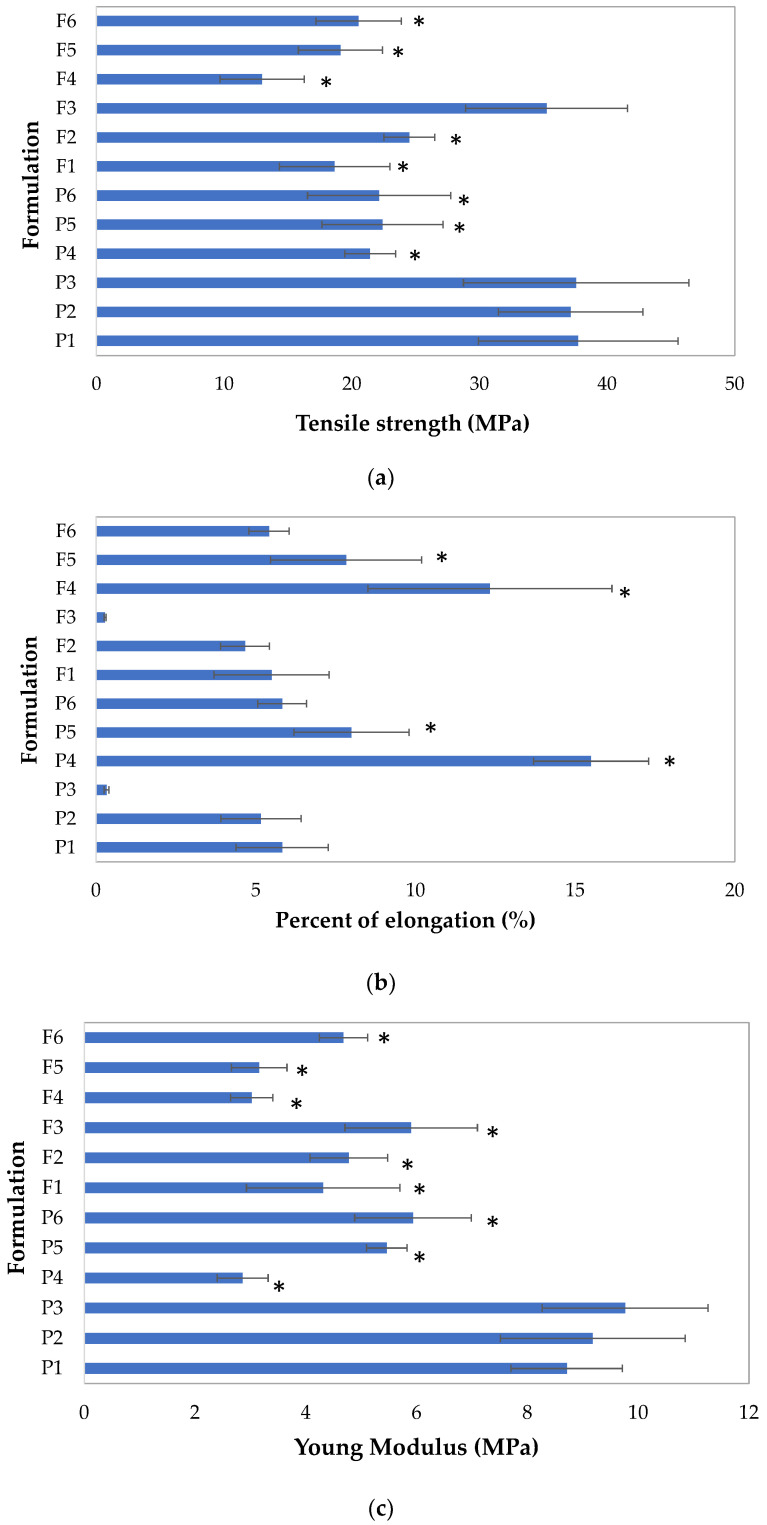
The mechanical properties of films placebo (P1–P6) and POS-loaded formulations (F1–F6) expressed as (**a**) tensile strength, (**b**) percent of elongation and (**c**) Young’s modulus (mean ± SD, *n* = 3, * significant differences (*p* < 0.05) in comparison to P1).

The mechanical characteristics of the prepared non-crosslinked and crosslinked films were analyzed by various criterion: tensile strength (TS), percent of elongation (E%),Young’s modulus (E) and folding endurance (Figure 6). The ideal films for buccal administration are strong, flexible films that are not susceptible to tearing or cracking during packaging or application of the drug formulation. More precisely, they should be characterized by a high folding endurance, E%, and low values of TS and E. In fact, hard and brittle films are characterized by higher values of E and TS, but elastic films possess higher E% values [3,17,28].

All prepared formulations were subjected to folding endurance evaluation. It was observed that all films had good mechanical properties and they withstood more than 100 folds. The values of the mechanical properties of the non-crosslinked and crosslinked ALG and ALG/PEC films are given in Figure 6. The TS values found ranged from 12.98 ± 3.30 MPa in formulation F4 to 37.73 ± 7.81 MPa in formulation P1. The highest values of E were found in the formulation P3 (9.76 ± 1.50 MPa), but formulation P4 was characterized by the lowest E (2.86 ± 0.46 MPa). It was shown that the ALG formulations were characterized by the highest TS and E values, which indicate that films based on the ALG are hard and brittle. PEC addition had a significant (*p* < 0.05) effect of reducing TS and E levels, but increased E%, which provides evidence that the films were more flexible. The crosslinking process slightly altered the TS values for the placebo formulations. However in POS-loaded films, Ca^2+^ presence significantly increased TS, increased E and decreased E%. In general, the mechanical properties of films based on the polymers depend on the chemical composition. ALG with a higher proportion of G units creates brittle, stiffer, but more stable gels. In contrast, ALG, which possesses in its structure high values of mannuronic (M) blocks, develops gels that are characterized by greater softness and flexibility. This fact was confirmed by Azeredo et al., who found that ALG gels that possessed high M-blocks content were characterized by notably more flexibility than formulations that possessed high G-blocks [29]. Moreover, the LM pectin gels show more flexible and branched network strands than purely HM pectin gels. Gohil concluded that, compared to the PEC and ALG/PEC films, ALG films have higher values of tensile strength. This fact might be related to their lower thickness and molecular structure [30]. Makaremi et al. also observed that ALG/PEC films were characterized by lower values of elongation at break [31]. Oakefull et al. investigated the interaction between ALG and PEC and suggested that polyguluronate blocks and the methylester region caused a rigid packed structure. They concluded that films containing ALG and PEC in a 1:1 ratio were more flexible than films composed from pure ALG [32]. Predictably, the received details represented that films subjected to the crosslinking process were characterized by notably higher durability and rigidity and decreased elasticity than non-crosslinked films, which showed high TS and E levels and reduced E% values. Similar arrangements were submitted by the Rhim, who noted that when ALG films were treated with CaCl_2_, TS values increased and E values decreased [33]. This fact might be related to conversion of the loose interactions between polymers for strong interactions created between the carboxyl groups of ALG and PEC and Ca^2+^ ions [34]. The E% of crosslinked ALG and PEC films were decreased, probably in relation to a crosslinking process setting in an aqueous solution, and in consequence, water was bounded in the interchain space. In addition, crosslinked films were characterized by higher values of thickness than non-crosslinked ones. This hypothesis was confirmed by Russo et al. who investigated the crosslinking process of ALG/polyglycerol by using Ca^2+^ ions and reported that the crosslinking reaction contributed to the reduction in chain mobility and, as a result, the reduction in flexibility [35].

It was concluded that placebo films were more brittle, but POS presence in the formulations influenced the reduction in the tested parameters: tensile strength (TS) (from 37.73 ± 7.81 MPa in formulation P1 to 18.66 ± 4.32 MPa in formulation F1), percent of elongation (E%) (from 15.15 ± 1.80% in formulation P4 to 12.33 ± 3.82% in formulation F4) and Young’s modulus (E) (from 9.76 ± 1.50 MPa in formulation P3 to 5.90 ± 1.19 MPa in formulation F3). In accordance with Shaw et al.’s research, the lower values of TS and the higher E% values might be related with the reduction in the number of intermolecular bonds between polymers, which leads to a reduction in all the parameters of mechanical properties [36]. Similar results were received by Gouveia et al., who recorded that the presence of choline chloride in the PEC formulations provided an impact of reducing tensile strength [37].

### 3.3. Swelling and Erosion

Polymer swelling is an important parameter, which has a high significance both in the process of mucoadhesion and in the drug release. Swelling of the polymer, initiating its deep contact with the mucus layer, ensures the formation of a spatial network capable of penetrating the mucin and, as a result, creating hydrogen and electrostatic interactions [38].

**Figure 7:**
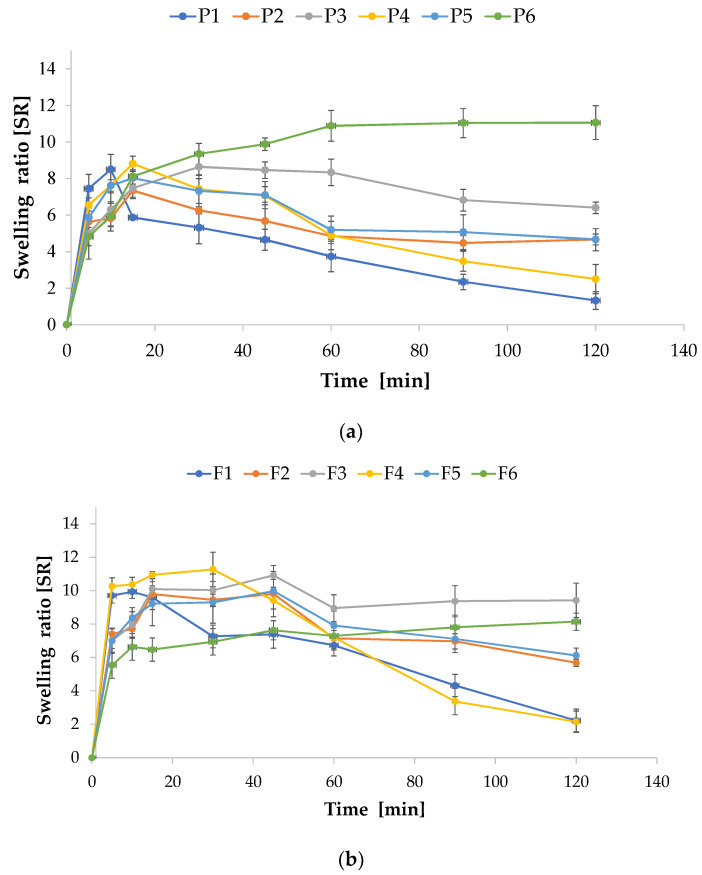
Swelling ratio (SR) of film formulations (**a**) without drug (P1–P6) and (**b**) containing POS (F1–F6) in SSS (simulated saliva solution, mean ± SD, *n* = 3).

Swelling features of the prepared non-crosslinked and crosslinked films were evaluated in the SSS as a function of time and expressed as swelling ratio (SR). The obtained results are depicted in Figure 7. It was noted that all prepared films exhibited swelling properties, which were different between formulations. It was found that the formulation containing only ALG was characterized by a maximum SR after 10 min (8.49 ± 0.83) and a gradual decline over the 120 min of the test. In turn, the formulation containing PEC (F4) reached a maximum of 8.81 ± 0.78 after 15 min. Similar reports were noticed by Silva et al. who concluded that films consisting of pure pectin exhibited the highest SR, reached after 20 min, compared with ALG films [39]. Günter et al. prepared ALG/PEC microparticles using an emulsion method containing PEC with a low degree of methylesterification, 36–44%. They reported that ALG/PEC microparticles were characterized by higher swelling in the pH of 6.8 than ALG microparticles [40]. Differences in the water absorption capacity of PEC are related to the molecular structure. SR is mainly influenced by the degree of esterification—when the degree of esterification is smaller, PEC has a greater capacity to absorb water [10]. It was shown that the crosslinked formulations were characterized by a higher SR (from 1.33 ± 0.48 in formulation P1 to 11.05 ± 0.92 in formulation P6 after 120 min). The swelling behavior of films was correlated with the erosion process (Table 5). It was shown that non-crosslinked formulations were eroded significantly, which resulted in a reduction in swelling degree. Generally, non-crosslinked ALG/PEC films were dissolved in about 2 h after immersion in SSS. While crosslinked formulations were characterized by the decreased erosion, they were more stable and absorbed water for a longer period of time with higher SR values compared to non-crosslinked formulations. The increased stability of these films can be attributed to the higher content of the Ca^2+^ ion, which provides an increased degree of crosslinking negatively charged galacturonic acid residues binding Ca^2+^ ions. The crosslinking process reduced water inflow into the polymer matrix, and consequently decreased the swelling ability in the first periods of the research [41]. In contact with the SSS, crosslinked films gradually released Ca^2+^, destabilizing the matrix, which leads to damage of the polymer network and water influx within the films. Additionally, in conditions with higher pH values, the polymer carboxylic acid residues were changed in carboxylate ions with a negative charge. This fact influenced the electrostatic repulsion between the polymer backbone and provided network spreading [42,43]. In addition, a reduction in the swelling capacity related with Ca^2+^ concentration was noted. Davidovich-Pinkas et al. suggested that a denser gel network, which is formed by the association of lateral chains, affected the reduced rate of diffusion [44]. Similar data were reported by Zactiti and Kieckbusch for ALG films with different degrees of crosslinking, where the swelling ability was notably reduced when Ca^2+^ concentration was increased [45]. When the Ca^2+^ ions’ concentration increased, the number of ALG groups increased, which might create an “egg-box” model, providing a stronger, crosslinked polymer network with lower water absorption capacity. Sriamornsak and Kennedy tested the swelling properties of ALG/PEC films crosslinked with Ca^2+^ and observed a higher swelling degree for PEC films compared to ALG films. This fact might be related to the higher number of crosslinking sites, which were responsible for more stable films characterized by low erosion [46]. In POS-loaded formulations, a considerable decrease in swelling ability was reported, which perhaps correlated with low POS solubility in SSS.

### 3.4. Mucoadhesion

**Figure 8:**
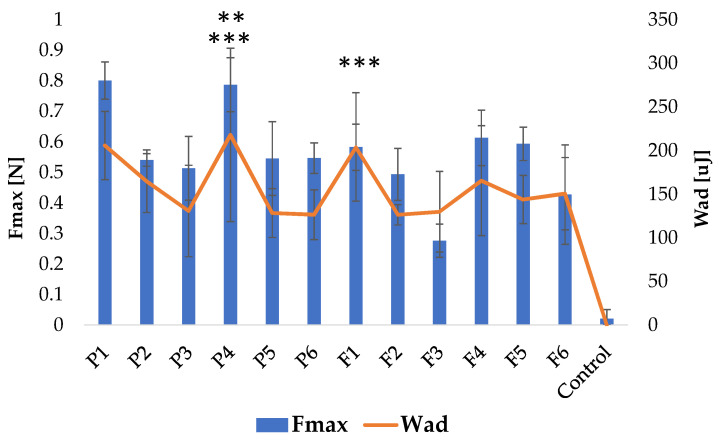
Mucoadhesive characteristics of formulated films without drug (P1–P6), formulations containing POS (F1–F6) and control (cellulose paper) (mean ± SD, *n* = 6; ** insignificant differences of F_max_ (*p* > 0.05) in comparison to P1; *** insignificant differences of W_ad_ (*p* > 0.05) in comparison to P1).

Mucoadhesive drug dosage formulations elongate the drug dosage form’s residence time at the target place, which might enhance its bioavailability, avoiding metabolic pathways. Additionally, mucoadhesive drug delivery systems are effective topical formulations that allow the use of a lower dose, reducing systemic exposure to the drug and reducing the possibility of side effects [47]. The mucoadhesiveness of the prepared films, depicted as the work of adhesion W_ad_ (μJ and detachment force F_max_ (N), was evaluated (Figure 8). An adhesive layer of the porcine buccal mucosa was applied to simulate the in vivo terms. It was noted that ALG and ALG/PEC films were characterized by good mucoadhesive properties with F_max_ from 0.276 ± 0.05 N in formulation F3 to 0.8 ± 0.06 N in formulation P1, and W_ad_ from 218.04 ± 99.43 μJ in P4 to 126.38 ± 28.57 μJ in P6 (Figure 8). The obtained results indicate that PEC presence slightly increases W_ad_ and F_max_ values. Jelvehgari et al. reported that when PEC content increased in ALG/PEC discs, adhesion also increased [48]. Comparable data were recorded by Lauren et al. who noted that PEC influenced the increase in the bioadhesion of metronidazole-loaded nanofibrillated cellulose films [49].

ALG is classified as an anionic mucoadhesive polymer, and it possesses a carboxyl group that forms hydrogen bonds with the hydroxyl groups of mucin glycoproteins. PEC molecules are also rich in carboxyl groups responsible for interactions with the mucin. In addition, the mucoadhesive PEC properties might result from the adsorption on the mucin layer. During contact between PEC and mucin, there is an increase in the negative charge because of the negative charge of PEC [50]. It was observed that the crosslinking process significantly reduced the mucoadhesive parameters (Figure 8). A higher degree of crosslinking formation of the more rigid film matrix reduced the flexibility of polymers, which leads to a decrease in the medium penetration and swelling properties, and, consequently, is responsible for reducing the mucoadhesive properties [51]. Similar results were obtained by Awasthi et al., who prepared dual crosslinked PEC/ALG beads with repaglinide and observed that the mucoadhesive properties decreased with an increasing degree of crosslinking [52]. In our study, it was also observed that POS-loaded formulations were characterized by a reduction in F_max_ and W_ad_ values compared to the placebo formulation. Similar details were noted by Pamlényi et al., who reported that the presence of cetirizine hydrochloride in ALG films reduced mucoadhesive properties [53].

**Figure 9:**
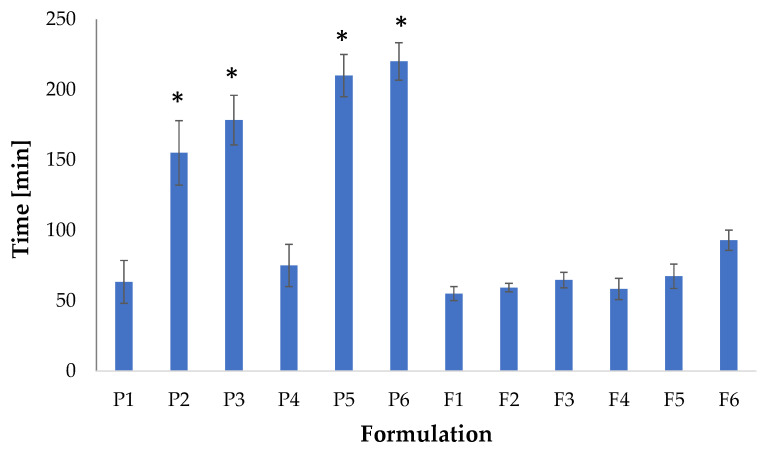
Ex vivo residence time of film placebos (P1–P6) and POS-loaded (F1–F6) formulations (mean ± SD, *n* = 3, * significant differences (*p* < 0.05) in comparison to P1).

Ex vivo retention time was estimated with porcine buccal mucosa and is expressed in Figure 9. It was noted that formulation F6 was characterized by the highest values of retention time, and it was 205.00 ± 10.00 min. The crosslinked film placebos possessed a higher retention time compared to the non-crosslinked formulations, which were characterized by lower stability and possessed a shorter disintegration time. The crosslinked formulations demonstrated a prolonged disintegration time, which affected the higher stability of the films in SSS and had a prolonged retention time. In addition, crosslinking formulations were characterized by a higher thickness, which prolonged the dissolution time. POS-loaded formulations possessed a lower retention time, which was correlated with the data obtained in the mucoadhesive test.

### 3.5. In Vitro Release

**Figure 10:**
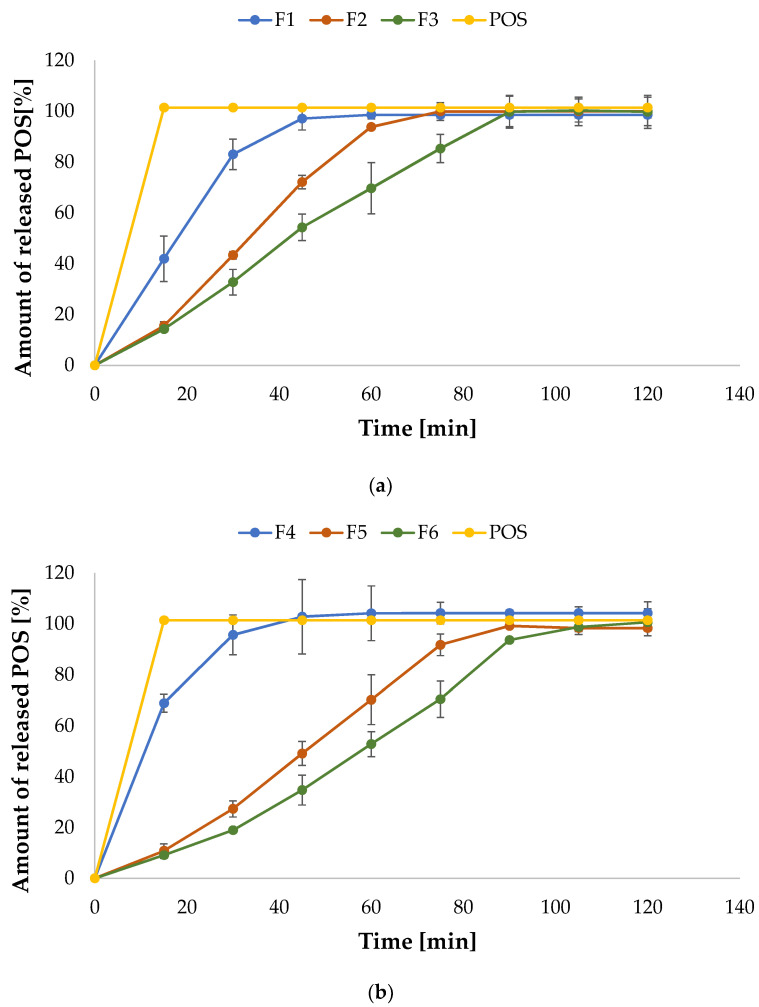
POS dissolution from (**a**) non-crosslinked formulations F1–F3 and (**b**) crosslinked formulations (F4–F6) and control (pure POS) (mean ± SD, *n* = 3).

The in vitro release profile analysis is not comparable with in vivo conditions, but it enables evaluation of the performance of the drug’s availability in the preliminary stages of product development, and it is an important part of the research of the quality of drug dosage forms [54]. The in vitro drug release profile is a crucial element, which impacts on the efficiency of substances with antifungal action, inhibition of pathogen growth and prevention of the development of drug resistance [55]. The release profile of POS was carried out in SSS as a dissolution medium with 1% SDS to maintain sink requirements. The obtained data are depicted in Figure 10. Despite the fact that POS is a substance that is practically insoluble in water, it was completely released within 2 h from all formulations. Drug solubility is a crucial parameter influencing the development of drug dosage formulations and one of the important factors influencing the bioavailability of therapeutic drugs. SDS is widely used in the pharmaceutical field as an ionic solubilizer and emulsifier applied in liquid and semi-solid formulations and as a lubricant in solid dosage forms. In addition, SDS is one of the most frequently used surfactants, enabling the maintenance of sink conditions [56,57]. The solubility of the compound in the amorphous form is greater than the crystalline form [58]. Therefore, the improvement in POS solubility might be the result of the active substance transition to the amorphous form as a result of the presence of a solubilizer (PEG 400), as suggested by the DSC test results (Section 3.7).

Comparing formulations F1 and F4, it was noted that the presence of PEC in the film formulation affected the faster release of POS. After 15 min, from F1, 41.92 ± 8.95% was released and from F4, 68.85 ± 3.53%, reaching the following values after 45 min—97.03 ± 4.48% and 102.75 ± 14.65%, respectively. This fact is related to the data received from swelling studies. SR has a fundamental influence in drug release studies—swelling causes the relaxation of polymer chains and enhances POS permeability from the film matrix. Formulation F4 was characterized by a higher SR, explaining the faster release of POS during the dissolution tests. A swollen matrix facilitates the inflow of water into the film and, as a result, leads to a faster release of the therapeutic substance [59]. Similar results were noted by Jaya et al. They reported that when the content of pectin increased, the acetylsalicylic acid release from ALG/PEC microcapsules also increased [60]. As expected, it was observed that both ALG and ALG/PEC crosslinked film formulations were characterized by the prolonged drug release. Similar results were reported by Awasthi et al., who observed sustained repaglinide release from dual crosslinked PEC/ALG beads [52]. POS release from crosslinked films containing PEC was slower than from crosslinked films containing only ALG, which was also confirmed by Awasthi et al. They concluded that Ca^2+^ crosslinked ALG/PEC beads were characterized by a more stable and more intact structure. In addition, they observed that the crosslinking process improved the mechanical resistance of the polymeric network and reduced swelling ability [52]. Crosslinked films possess a more rigid structure as a result of higher chain entanglement, which hindered drug release during the swelling, and prolonged POS release was observed. PEC, especially low methoxy (LM) PEC (with DM <50%) after contact with Ca^2+^ ions, creates a strong gel as a result of the crosslinking of the galacturonic acid chains [60]. A higher Ca^2+^ ions quantity enables a higher degree of polymer crosslinking, which reduces the water influx and consequently extends the release of the drug. This fact was confirmed by Sungthongjeen et al., who reported that increasing the calcium content in the PEC matrix tablets resulted in a prolonged release profile of indomethacin [61].

The mechanism of POS release from non-crosslinked and crosslinked ALG and ALG/PEC films was established by analyzing various models. For the assessment, the zero-order kinetics, first-order kinetics, Higuchi, Hixson-Crowell and Korsmeyer–Peppas models were applied (Table 6). It was noted that from all tested formulations, POS was released according to zero-order kinetics, which was characterized by the highest curve linearity. In accordance with the Korsmeyer-Peppas equation, values of the *n* parameter were below 0.5 and reached from 0.09 in formulation F1 to 0.17 in formulations F2 and F5 [19]. This fact implied that the mechanism of POS release was a Fickian diffusion. This was confirmed by data obtained from the Higuchi model. Hence, a high linearity from 0.88 to 0.99 was observed in the Hixson-Crowell model and testifies to the fact that POS release was related to the connection of the diffusion and the film’s erosion, which provides the gradual dissolution of the film matrix in the medium. This is in agreement with the results published by Ramos et al. [62], who examined a hydroalcoholic extract of *Macrocystis pyrifera* released from ALG films.

### 3.6. Antifungal Activity

**Figure 11:**
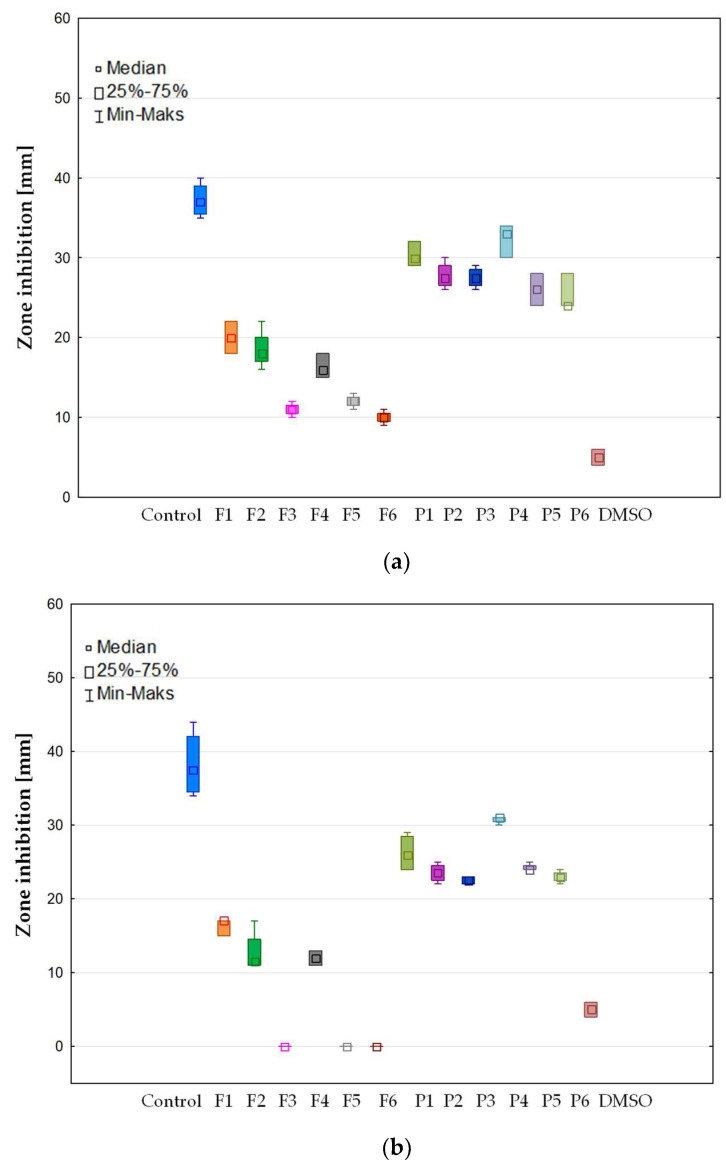

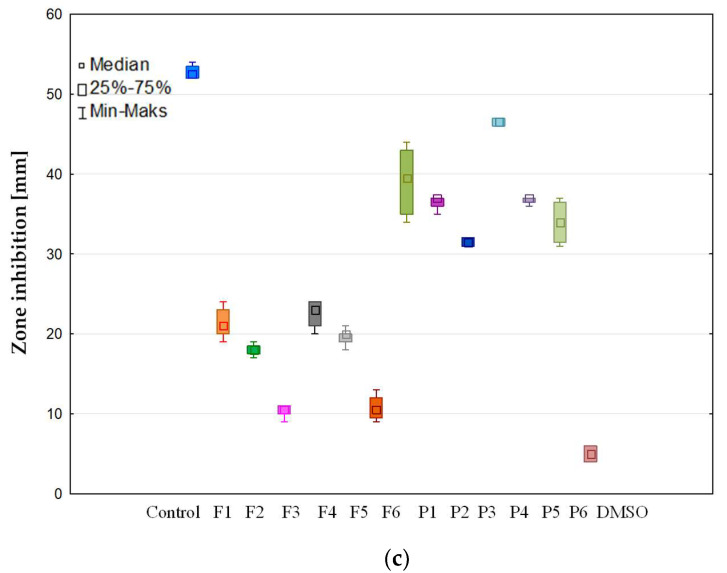
Antifungal action of film placebo formulations (P1–P6) and formulations containing POS (F1–F6); control (POS in DMSO) toward (**a**) *C. albicans*, (**b**) *C. krusei* and (**c**) *C. parapsilosis* (*n* = 6).

In this work, the antifungal activity of non-crosslinked and crosslinked films on *Candida* spp. was assessed. The test was conducted based on the CLSI regulations [20]. Antifungal action was verified following incubation for 24–48 h and expressed as the mean diameter (mm) of the zone of inhibition growth (Figure 11). It was noted that all the prepared film formulations inhibited the growth of *Candida albicans* and *Candida parapsilosis*. The highest antifungal activity of the prepared films was noted in the case of *Candida parapsilosis*, and this was from 10.33 ± 0.82 mm (formulation P3) to 46.50 ± 0.58 mm (formulation F4). In the case of the *Candida krusei* strain, it showed the lowest antifungal activity of all tested formulations (Figure 11b). In addition, formulations P3, P5 and P6 did not have any activity against this species. Comparing the placebo formulations, it was shown that the highest antifungal activity possessed the P1 formulation, which was composed from ALG only. However, in the case of POS-loaded formulations, the formulation composed of ALG and PEC (F4) in non-crosslinked films was characterized by the strongest antifungal effect, and this was from 30.75 ± 0.50 mm in *Candida krusei* to 46.50 ± 0.58 mm in *Candida parapsilosis*. Interestingly, the non-crosslinked formulation possessed antifungal activity against all tested *Candida* strains but for the crosslinked formulation, this was only against *Candida albicans* and *Candida parapsilosis.* This fact is related to the polymer’s antifungal activity—there are reports that ALG is characterized by a promising antimicrobial effect per se [6]. The antifungal ALG mechanism might be related to the negative charge of the polymer, which interacts with the surface of the fungal cell and leads to disruption of the cell wall. Moreover, the viscous polymer layer as a result of swelling leads to an impediment of nutrient uptake by the fungus cell. The antimicrobial ability might also be combined with the ALG’s ability to chelate ions, which reduce the production of metal-dependent proteins, limiting cell growth [63,64].

It was also shown that PEC’s addition increased the antifungal activity of the films. This fact might be related with the higher swelling properties of ALG/PEC films, which affect the faster release of POS and, as a result, greater antifungal activity. However, surprising antimicrobial PEC properties were reported in the 1990s. PEC is referred to as only food fiber, which possesses broad-spectrum antimicrobial properties against Gram-negative bacteria, yeasts and non-filamentous fungi and inactivates therapeutic bacteriophages at a high concentration (>2%) [65]. The mechanism of PEC antimicrobial action involves the ability to bind and destroy the outer membrane of bacterial or fungi cells [66]. It was reported that PEC with a minimum inhibitory concentration (MIC) of 40 mg/mL reduced *Staphylococcus aureus* ATCC 25,923 growth, with an MIC of 0.162 mg/mL—Gram-negative *Helicobacter pylori*, and with an MIC of 25 mg/mL—Escherichia *coli*. In addition, there are reports that the 1% PEC solution is characterized by antifungal activity against *Candida albicans* and *Saccharomyces cerevisiae* [65].

It was noted that the crosslinking process had a significant effect on the decrease in the antifungal activity. This is probably related to lower swelling properties and the prolonged release of POS.

### 3.7. Thermal Analysis

Generally, thermal analysis is a term describing an analysis technique of the physical changes occurring in the tested samples over time under the influence of temperature [67]. Thermal analysis is often applied in pharmaceutical technology to assess quality, thermal stability and potential interactions between components of drug formulations. Therefore, all presented formulations as well as the excipients were analyzed by thermal methods: thermogravimetry (TGA) and differential scanning calorimetry (DSC).

**Figure 12:**
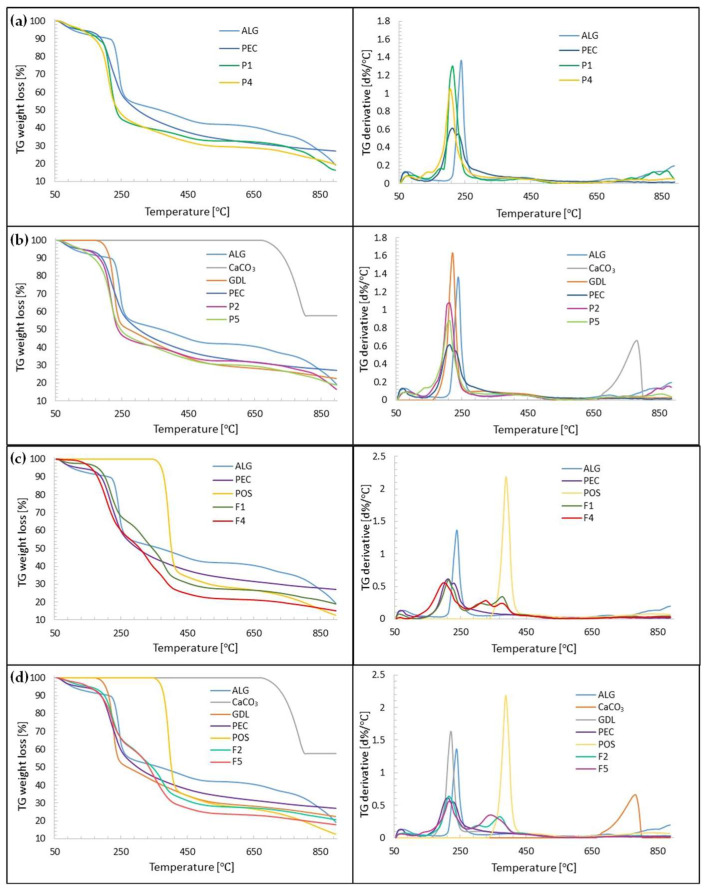
TGA curves of film placebo formulations: (**a**) non-crosslinked P1 and P4, (**b**) crosslinked P2 and P5; and POS-loaded formulations: (**c**) non-crosslinked F1 and F4, (**d**) crosslinked F2 and F5 and their components.

Thermogravimetric analysis (TGA) provides details regarding thermal stability and the behaviour of samples at elevated temperatures. The analysis relies on examining variations in the mass of the test substance during a gradual increase in the temperature at a constant rate. In the TGA curves, a slight weight loss up to 150 °C was observed in all formulations, which represented the evaporation of moisture, volatiles and residual solvent (Figure 12). The placebo formulations, P1, P2, P4 and P5, decomposed in one significant step in terms of temperatures 150 °C and 320 °C (about 50% weight loss for all formulations). All formulations further decomposed in the temperature range between 320 °C and 900 °C, demonstrating 24, 20, 24 and 21% weight loss for formulations F1, F4, F2 and F5, respectively, corresponding to the ALG content of these formulations. The maximum decomposition rate was at 215 °C for P1, 205 °C for P4 and 215 °C for P2 and P5. In all formulations, thermal decomposition began at lower temperatures than for the pure excipients. In the TGA curves, a slight weight loss up to 150 °C was observed in all formulations, which represented the evaporation of moisture, volatiles and residual solvent. Formulations F1, F2, F4 and F5 loaded with POS decomposed in two stages. The first stage covered the temperature range from 150 °C to 250 °C (showing about 31% weight loss for formulations F1, F2 and F5, and 35% weight loss for formulation F4). The second stage of decomposition was recorded in the temperature range between 250 °C and 450 °C with maximum decomposition rates at 370 °C (F2), 380 °C (F1 and F4) and 345 °C (F5) and corresponded to POS decomposition. All formulations further decomposed slightly in the temperature range between 450 °C and 900 °C, demonstrating 13% and 11% weight loss for formulations F1 and F2, respectively, and 10% weight loss for F4 and F5. In all formulations, thermal decomposition began at lower temperatures than for the pure excipients.

**Figure 13:**
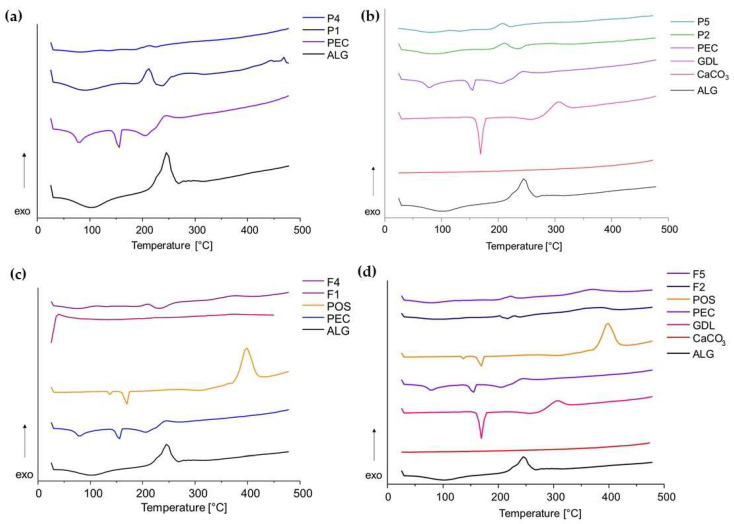
DSC thermograms of film placebo formulations: (**a**) non-crosslinked P1 and P4, (**b**) crosslinked P2 and P5, and POS-loaded formulations: (**c**) non-crosslinked F1 and F4, (**d**) crosslinked F2 and F5 and their components.

DSC thermograms of formulations without a drug, P1, P2, P4 and P5, and formulations containing POS, F1, F2, F4, F5, and their components are presented in Figure 13. The ALG DSC curve introduced a wide endothermic peak in the range of 48.33 °C–165.03 °C and a sharp exothermic peak at 244.76 °C. The broad endothermic peak might be related with the removal of three various forms of water linked with ALG: free released (in the temperature range 40 °C–60 °C), interacting with ALG hydroxyl groups (eliminated around 120 °C) and associated with ALG carboxylic acid groups (at 160 °C) [68]. The exothermic peak detected at 244.76 °C in the polymer proved the polymer’s decomposition. Dudek et al. suggested that this peak was the result of the processes of dehydration, depolymerization and destruction of the ALG saccharide ring [69]. The ALG glass transition is present from 100 °C to 150 °C, but the broad endothermic peak is masked. The PEC thermogram showed three endothermic peaks. The first was noted up to 82.98 °C, and it is related with the loss of water, which was bounded with the polymer. A further expressive endothermic peak was noted at 155.55 °C, and it was probably connected with the conversion of the galacturonan ring chair conformation to the inverse chair conformation. At 244.42 °C the exothermic peak was observed, which is correlated with PEC thermal degradation followed by the elimination of volatile products [70]. PEC degradation was primarily derived from pyrolytic decomposition and decarboxylation pathways [71].

The DSC thermogram of GDL presented an endothermic peak at 169.28 °C, which is related with the melting point. In addition, GDL possessed an endothermic peak at 309.93 °C, which demonstrated its decomposition. The DSC of the CaCO_3_ curve depicts a straight line because its melting point is at about 825 °C [72]. At 169.52 °C, a sharp endothermic peak was noted, which indicated the melting point of unprocessed POS. Similar detail was expressed by Hens et al. [73]. The received data demonstrate that the POS crystalline character was maintained. The decomposition of POS, during one stage, was indicated at a temperature ranging from 370.45 °C to 431.26 °C, with the maximum at 399.09 °C.

The DSC curves of placebo formulations exhibit a light shift and the reduction in exothermic polymer peaks. In crosslinking formulations, the GDL decomposition peak was not observed. In DSC thermograms of POS-loaded formulations, peaks of ALG, POS and GDL were not observed, indicating that those excipients and drugs are present in the formulation in their amorphous forms. The absence of a POS melting point might be the result of the transition of the active substance to its amorphous form with regard to the solubilizer’s addition (PEG 400). It is known that amorphous substances are characterized by higher solubility, stability and bioavailability than their crystalline forms [58].

### 3.8. Attenuated Total Reflectance–Fourier Transform Infrared Spectroscopy (ATR–FTIR)

Attenuated total reflectance–Fourier transform infrared spectroscopy (ATR–FTIR) is an analytic method used to evaluate the construction of particular molecules and the constitution of complex chemical compounds by modulated mid-infrared energy. At certain frequencies, infrared light is absorbed by the molecule, and the bonds within it vibrate at different frequencies in a characteristic manner, creating a “fingerprint” of the molecule. FTIR–ATR allows the detection of interactions between the drug and polymer utilizing signals of functional groups and details about their bands—changes, frequency, shifts and intensities [74].

**Figure 14:**
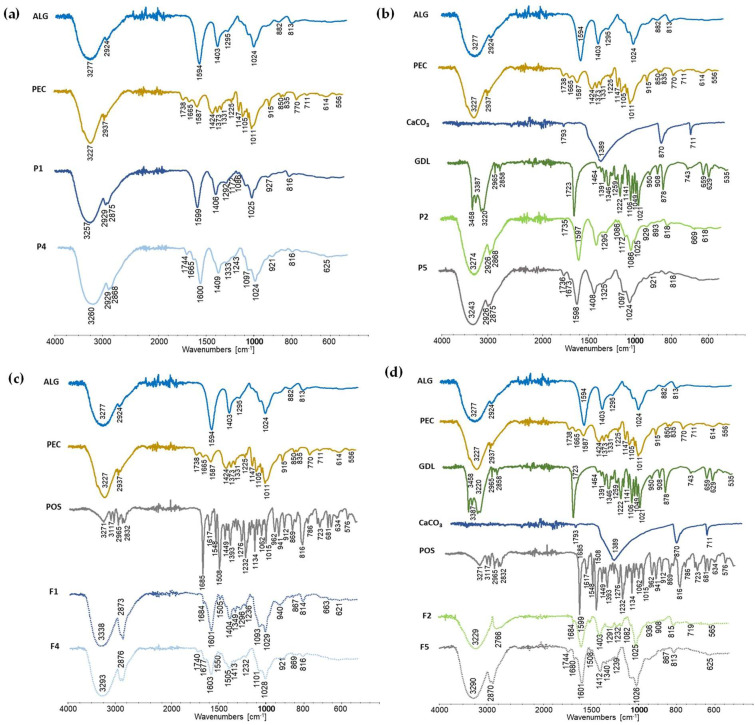
FTIR-AR spectra of film placebo formulations: (**a**) non-crosslinked P1 and P4, (**b**) crosslinked P2 and P5, and POS-loaded formulations: (**c**) non-crosslinked F1 and F4, (**d**) crosslinked F2 and F5 and their components.

FTIR–ATR spectra of ALG, PEC, CaCO_3_, POS, formulations without drugs (P1, P2, P4, P5) and formulations containing POS (F1, F2, F4, F5) are presented in Figure 14. There were a wide band of O-H bonds stretching vibrations with a maximum at 3257 cm^−1^, and a signal from C-H bonds stretching vibrations at 2929 cm^−1^ observed in the ALG spectrum. Furthermore, a band of asymmetric vibrations (1599 cm^−1^), and a band of symmetric vibrations (1406 cm^−1^) originating from carboxylic salt were present. The stretching vibrations of C-O bonds present in the pyranose ring (1025 cm^−1^) were also noted. Spectra of the physical mixtures were dominated by signals originating from ALG, PEC and GDL. In the spectra of formulations containing PEC (formulation placebos P4, P5 and POS-loaded formulations F4, F5), signals at 1665 cm^−1^, which represent amide bond vibrations, were presented. In the crosslinked formulations, GDL signals were noted. GDL bands that originated from C=O bonds vibrations were observed at 1735 cm^−1^—characteristic of lactone. However, signals coming from the vibrations of CaCO_3_ bonds were not visible. This fact might be related with the low concentration of CaCO_3_ in drug formulation. The FTIR–ATR spectra of substances in low concentrations might not be detectable—the method will show the signals of components that are present in the formulation at a level of at least 1% [75]. Furthermore, in spectra representing POS-loaded formulations (F1, F2, F4, F5), gentle signals characteristic of bond vibrations presented in POS were observed, as well as characteristic C=O vibration signals from the urea functional group at round 1680 cm^−1^, aromatic ring stretch (1509 cm^−1^), C-N bonds stretch (1349 cm^−1^), C-O aryl-alkyl ether vibrations (1232 cm^−1^), C-F stretching vibrations (1093 cm^−1^) and aromatic ring out of plane bend (867 cm^−1^). The presence of these characteristic signals indicates good compatibility of a drug with excipients.

## 4. Conclusions

The purpose of this research was to apply a one-step crosslinking method to prepare ALG and ALG/PEC buccal films with posaconazole—by using CaCO_3_ and GDL. The presence of PEC in the ALG films improved flexibility and mucoadhesion and the antifungal activity of the developed formulations. The application of a crosslinking agent led to stronger, stiffer and water-resistant ALG/PEC films being obtained, which possessed a lower tensile strength, percent of elongation and Young’s modulus than non-crosslinked formulations. It was observed that the crosslinking process reduced mucoadhesiveness and antifungal activity against tested *Candida* spp. It was concluded that formulation F5 consisting of ALG and PEC and crosslinked by using CaCO_3_ and GDL in a 0.05:0.19 ratio was characterized by optimal mucoadhesive, mechanical and antifungal properties, and prolonged posaconazole release.

## Figures and Tables

**Table 1:** Composition of film formulation placebos (P1–P6) and film POS-loaded formulations (F1–F6).

Formulation	ALG (g)	PEC (g)	GLY (g)	PEG (g)	POS * (g)	CaCO_3_ (g)	GDL (g)	Purified Water (up to)
P1	2	–	1	1	–	–	–	100
P2	2	–	1	1	–	0.05	0.19	100
P3	2	–	1	1	–	0.07	0.214	100
P4	1	1	1	1	–	–	–	100
P5	1	1	1	1	–	0.05	0.19	100
P6	1	1	1	1	–	0.07	0.214	100
F1	2	–	1	1	0.649 *	–	–	100
F2	2	–	1	1	0.649 *	0.05	0.19	100
F3	2	–	1	1	0.649 *	0.07	0.214	100
F4	1	1	1	1	0.649 *	–	–	100
F5	1	1	1	1	0.649 *	0.05	0.19	100
F6	1	1	1	1	0.649 *	0.07	0.214	100

* to provide 2 mg POS/cm^2^.

**Table 2:** Viscosity and pH of the ALG and ALG/PEC non-crosslinked solutions and crosslinked hydrogels (*n* = 3).

Formulation	Viscosity (mPa∙s)	pH
P1	3164.02 ± 83.23 *	7.82 ± 0.01 *
P2	5137.39 ± 982.41 *	6.44 ± 0.01 *
P3	21,486.64 ± 701.85 *	6.39 ± 0.02 *
P4	650.44 ± 19.10 *	4.92 ± 0.01 *
P5	13,808.12 ± 1187.72 *	4.79 ± 0.04 *
P6	23,548.21 ± 1018.86 *	4.77 ± 0.02 *
F1	3197.09 ± 68.85 *	8.02 ± 0.02 *
F2	8610.09 ± 793.99 *	6.72 ± 0.03 *
F3	25,786.17 ± 726.36 *	6.54 ± 0.01 *
F4	760.69 ± 33.08 *	5.00 ± 0.02 *
F5	17,385.55 ± 687.68 *	4.96 ± 0.03 *
F6	27,131.16 ± 1490.99 *	4.89 ± 0.02 *

* statistically significant difference (*p* < 0.05).

**Table 3:** Assessment of non-crosslinked and crosslinked film formulations (mean ± SD, *n* = 3).

Formulation	Thickness (μm)	Surface pH	Moisture Content (%)	Weight Uniformity (mg)	Drug Content (mg)
P1	57.67 ± 5.19 *	6.94 ± 0.02	4.39 ± 1.49 *	38.28 ± 3.43 *	–
P2	61.56 ± 7.16 *	6.91 ± 0.02	5.71 ± 1.20	41.93 ± 0.39 *	–
P3	65.28 ± 3.80 *	6.91 ± 0.02	5.19 ± 1.37	40.55 ± 4.49	–
P4	58.94 ± 4.60 *	6.90 ± 0.03	5.11 ± 1.13	40.63 ± 1.99	–
P5	60.44 ± 5.90 *	6.89 ± 0.01	5.15 ± 1.73	40.25 ± 0.70	–
P6	68.17 ± 5.42 *	6.89 ± 0.02	5.36 ± 1.70	43.50 ± 4.15 *	–
F1	83.06 ± 9.31 *	6.91 ± 0.02	4.79 ± 1.04 *	52.98 ± 2.45 *	106.39 ± 6.05 *
F2	99.61 ± 9.45 *	6.89 ± 0.01	4.87 ± 0.97 *	56.74 ± 1.97 *	99.27 ± 1.86
F3	110.39 ± 5.15 *	6.88 ± 0.01	4.11 ± 0.85 *	57.88 ± 3.62 *	97.79 ± 2.19 *
F4	122.28 ± 9.80 *	6.88 ± 0.01	5.19 ± 1.69	52.34 ± 5.48 *	99.92 ± 3.29
F5	128.11 ± 9.71 *	6.87 ± 0.02	5.01 ± 1.34	56.38 ± 3.20 *	95.11 ± 4.43 *
F6	139.22 ± 3.72 *	6.87 ± 0.01	5.03 ± 1.36	57.32 ± 5.13 *	94.72 ± 4.01 *

* significant differences (*p* < 0.05).

**Table 4:** Disintegration time of prepared non-crosslinked and crosslinked films (mean ± SD, *n* = 3).

Formulation	Disintegration Time (min)	
	Conventional Apparatus	On Petri Dish
P1	2.23 ± 1.21	55 ± 10
P2	5.67 ± 0.94 *	>300 *
P3	7.25 ± 1.11 *	>300 *
P4	3.97 ± 1.04	125 ± 10 *
P5	7.33 ± 0.82 *	>300 *
P6	9.02 ± 0.79 *	>300 *
F1	3.35 ± 0.75	105 ± 10 *
F2	5.66 ± 1.17 *	>300 *
F3	8.09 ± 1.04 *	>300 *
F4	4.65 ± 1.46	125 ± 10 *
F5	8.53 ± 0.54 *	>300 *
F6	10.68 ± 1.63 *	>300 *

* significant differences (*p* < 0.05) in comparison to formulation P1.

**Table 5:** Erosion tests of film formulations without drug (P1–P6) and containing POS (F1–F6) in SSS (simulated saliva solution, mean ± SD, *n* = 3).

Formulation	Erosion
P1	49.16 ± 6.85
P2	19.93 ± 0.87 *
P3	6.69 ± 3.47 *
P4	51.70 ± 6.98
P5	6.30 ± 3.01 *
P6	3.07 ± 1.00 *
F1	61.88 ± 5.05
F2	29.43 ± 4.98 *
F3	12.53 ± 4.28 *
F4	62.17 ± 12.02
F5	12.02 ± 1.31 *
F6	11.64 ± 3.28 *

* significant differences (*p* < 0.05) in comparison to P1.

**Table 6:** Models of POS release from ALG and ALG/PEC films.

Formulation	Zero Order Kinetics		First Order Kinetics		Higuchi Model		Hixson-Crowell Model		Korsmeyer-Peppas Model		
	R^2^	K	R^2^	K	R^2^	K	R^2^	K	R^2^	K	*n*
F1	0.81	1.23	0.76	0.02	0.88	14.87	0.94	4.59	0.84	0.17	0.09
F2	0.96	1.46	0.85	0.29	0.98	18.63	0.97	5.64	0.92	0.06	0.17
F3	0.97	1.01	0.85	0.02	0.99	14.57	0.93	5.64	0.93	0.07	0.13
F4	0.79	0.75	0.76	0.01	0.86	9.15	0.92	4.77	0.84	0.16	0.05
F5	0.99	1.25	0.89	0.03	0.98	16.81	0.95	5.53	0.96	0.02	0.17
F6	0.97	0.98	0.89	0.02	0.97	14.76	0.89	5.95	0.96	0.02	0.15

R^2^: correlation coefficient, K: release constant and *n*: the release exponent.

## Data Availability

Data are contained within the article; raw data are available upon request.
